# Phytochemicals and Gastrointestinal Cancer: Cellular Mechanisms and Effects to Change Cancer Progression

**DOI:** 10.3390/biom10010105

**Published:** 2020-01-08

**Authors:** Raghad Khalid AL-Ishaq, Anthony J. Overy, Dietrich Büsselberg

**Affiliations:** Department of Physiology and Biophysics, Weill Cornell Medicine-Qatar, Education City, Qatar Foundation, Doha 24144, Qatar; rkmalishaq@hotmail.com (R.K.A.-I.); ajo2001@qatar-med.cornell.edu (A.J.O.)

**Keywords:** phytochemical, gastrointestinal cancer, intestinal cancer, apoptosis, anti-cancerous effects

## Abstract

Gastrointestinal (GI) cancer is a prevailing global health disease with a high incidence rate which varies by region. It is a huge economic burden on health care providers. GI cancer affects different organs in the body such as the gastric organs, colon, esophagus, intestine, and pancreas. Internal and external factors like smoking, obesity, urbanization, genetic mutations, and prevalence of *Helicobacter pylori* and Hepatitis B and Hepatitis C viral infections could increase the risk of GI cancer. Phytochemicals are non-nutritive bioactive secondary compounds abundantly found in fruits, grains, and vegetables. Consumption of phytochemicals may protect against chronic diseases like cardiovascular disease, neurodegenerative disease, and cancer. Multiple studies have assessed the chemoprotective effect of selected phytochemicals in GI cancer, offering support to their potential towards reducing the pathogenesis of the disease. The aim of this review was to summarize the current knowledge addressing the anti-cancerous effects of selected dietary phytochemicals on GI cancer and their molecular activities on selected mechanisms, i.e., nuclear factor kappa-light-chain-enhancer of activated B cells (NF-κB), detoxification enzymes, adenosine monophosphate activated protein kinase (AMPK), wingless-related integration site/β-catenin (wingless-related integration site (Wnt) β-catenin, cell apoptosis, phosphoinositide 3-kinases (PI3K)/ protein kinase B AKT/ mammalian target of rapamycin (mTOR), and mitogen-activated protein kinase (MAPK). In this review phytochemicals were classified into four main categories: (i) carotenoids, including lutein, lycopene, and β-carotene; (ii) proanthocyanidins, including quercetin and ellagic acid; (iii) organosulfur compounds, including allicin, allyl propyl disulphide, asparagusic acid, and sulforaphane; and (iv) other phytochemicals including pectin, curcumins, p-coumaric acid and ferulic acid. Overall, phytochemicals improve cancer prognosis through the downregulation of β-catenin phosphorylation, therefore enhancing apoptosis, and upregulation of the AMPK pathway, which supports cellular homeostasis. Nevertheless, more studies are needed to provide a better understanding of the mechanism of cancer treatment using phytochemicals and possible side effects associated with this approach.

## 1. Gastrointestinal Cancer and Phytochemicals

### 1.1. Gastrointestinal Cancer

Cancer is a leading cause of death worldwide, being responsible for approximately 7.9 million deaths (13% of all deaths) [1]. The rate of cancer-related death is expected to rise to an estimated 12 million deaths by 2030 [2]. Gastrointestinal cancer (GI) is the second most common cause of cancer-related death in the world [3]. Statistical results obtained in 2008 showed that GI cancer is the fourth most common cancer in men and the fifth most common cancer in women [4]. GI cancer is a malignant condition which affects the gastrointestinal tract and accessory organs such as the colon, esophagus, and intestine [5]. The carcinogenesis of GI cancer occurs due to the accumulation of genetic variation of multiple genes such as tumor suppressors, mismatch repair genes, and oncogenes [6]. Imbalance between cellular proliferation and apoptosis leads to the pathogenesis of GI cancer [7]. Internal and external factors such as genetic, obesity, alcohol consumption, and *Helicobacter pylori* infection contribute to the pathogenesis of GI cancer [8]. Although patients with GI cancer become symptomatic after they have advanced lesions with either local or distant metastasis, commonly presented findings include bloating, epigastric pain, and palpable epigastric mass [9]. Though the incident rate of GI cancer is declining, it remains a major health problem and a huge burden on health care providers [10]. The prognosis of GI cancer is variable between patients depending on its progression at the time of detection. Early detection of GI cancer improves the outcomes of patients. Treatments of the disease include surgery, radiation, and administration of chemotherapy components such as cisplatin, mitomycin, and docetaxel injection [11].

### 1.2. Colorectal Cancer

Colorectal cancer (CRC) is the fourth most common malignant tumor in the world, with an incidence of 1.2 million new cases and over 600,000 death cases [12]. CRC is the second most common cancer in women and the third most common cancer in men worldwide [10]. As CRC is a so-called westernized disease, the highest incidence rates are found in Australia, New Zealand, North America, and Europe [13]. Although advance treatments are available to improve the survival rate of the disease, CRC remains an incurable disease [14]. While the rate of CRC in adults aged 50 and above decreases, an increase in disease incidence is observed in adults younger than 50 [15]. This suggest that factors such as physical activity, gut microbiome composition, and diet may underline the development of the disease [16]. Like most cancers, CRC is driven by an accumulation of genetic mutations in tumor suppressors such as adenomatous polyposis coli (APC), Smad4 and p53, and oncogenes such as K-ras [17]. These mutagenic accumulations lead to a stepwise progression from normal intestinal epithelial cells to pre-malignant tumor development/adenoma to adenocarcinoma [18]. Etiologically, CRC may be sporadic (more than 80% of cases are sporadic), hereditary, or be related to a history of inflammatory bowel disease [19]. Signs of colon cancer include change in bowel dietary habits and blood in stools [20]. Although treatment of CRC depends on the time of diagnosis and the stage of the disease, common treatments used include surgery, radiation, immunotherapy, and chemotherapy [21].

### 1.3. Esophageal Cancer

Esophageal cancer is a serious malignancy which accounted for more than 400,000 deaths worldwide in 2005 [22]. Although the incidence rate of other types of cancer is expected to decrease by 2025, the prevalence of esophageal cancer is expected to increase by 140% [23]. The two predominant histological subtypes of esophageal cancer are adenocarcinoma and squamous cell carcinoma, with these having unreliable racial and geographical distribution [24]. Although squamous cell carcinoma remains the most common type of esophageal cancer globally, adenocarcinoma has become the leading type in Western countries due to the higher incidence of obesity and Barrett’s esophagus [25]. Treatment of esophageal cancer includes surgery, radiation, and chemotherapy [26].

### 1.4. Diet and Microbial Metabolites

The gastrointestinal tract in the human body has the highest population of different microbes, such as in the microbiome. They play a critical role in the well-being of the host [27]. It is estimated that the human gut contains between 30 trillion to 400 trillion micro-organisms [28]. The interaction between the microbiome with different parts of the human gut (mucus layer, epithelial cells, and immune cells) helps in determining the health or disease status of the host [29]. Changes in the gut microbiota due to environmental exposure, host genetics, and diet are known to affect human physiology, prevalence of disease, and nutrition [30]. The gut composition of people lacking *Helicobacter pylori* infection has identified 128 phylotypes within 8 bacterial phyla of which Proteobacteria, Firmicutes, Bacteroidetes, Fusobacteria, and Actinobacteria are the most abundant [31]. Epidemiological studies have indicated that a diet with high fiber and low red meat and fat content reduces the risk of CRC due to the presence of colonic microbiota [32]. They enhance the host’s health by promoting the metabolism of fiber to produce short chain fatty acids (SCFAs) such as butyrate which downregulate pro-inflammatory cytokines such as interleukin-6 (IL-6) and interleukin-12 (IL-12) [33].

### 1.5. Impact of Gastrointestinal Cancer on Selected Pathways

**Figure 1:**
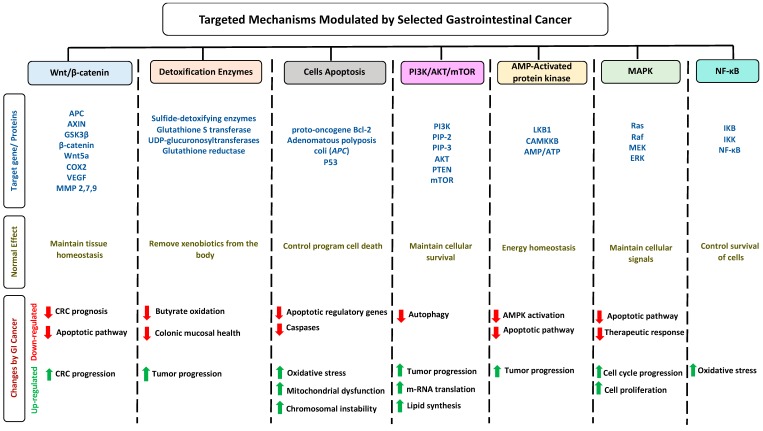
Schematic illustration of seven selected mechanisms modulated by GI cancer. The figure is divided into seven columns and three rows. The column headings represent the pathways while the row headings represent target genes/proteins for each pathway (blue), the overview physiological effect of these genes on pathways (dark yellow), and changes occurring on these pathways modulated by GI cancer.

Cancer has become one of the leading causes of death due to the difficulty of treating the disease [34]. Complications of cancer can be divided into direct and indirect complications, such as invasion and immune suppression, respectively [35]. Cancer mutations affect several mechanisms and pathways in the body (Figure 1). The wingless-related integration site (Wnt) transduction signaling pathway is an important mediator to maintaining repair and tissue homeostasis [36]. In patients with GI cancer, the phosphorylation of β-catenin increases, reducing the apoptotic pathway in the body [37]. The expression of detoxification enzymes decreases during cancer, thus reducing butyrate expression [38]. GI cancer enhances the activation of apoptotic pathways by reducing the activity of apoptotic regulatory genes and caspases that upregulate oxidative stress, mitochondrial dysfunction, and chromosomal instability [39]. In addition, the phosphoinositide 3-kinases (PI3K)/AKT/mTOR intracellular pathway is activated during GI cancer; this pathway upregulates tumor progression, lipid synthesis, and m-RNA translation [40]. Adenosine monophosphate activated protein kinase (AMPK) plays a multiple beneficial role in gut health; it improves barrier function and intestinal absorption, suppresses colorectal carcinogenesis, and reduces intestinal inflammation [41]. The deactivation level of AMPK pathways reduces during GI cancer and as a result enhances tumor progression and reduces apoptotic pathway activation [42]. Nuclear factor kappa-light-chain-enhancer of activated B cells (NF-κB) and mitogen-activated protein kinase (MAPK) pathways reduce during GI cancer, potentially leading to enhanced cell cycle progression, oxidative stress, and cellular proliferation [43,44]. High levels of NF-κB activity in early gastric carcinoma may enhance the prognosis of the disease [45]. Ninety-two genes have been tested to evaluate the relationship between NF-κB and CRC. The results have shown that out of 92 genes, 22 genes are significantly downregulated while nine genes are significantly upregulated, suggesting a strong correlation between NF-κB and colorectal cancer [46]. It has been shown that the inhibition of the NF-κB pathway leads to the reduction in vascular endothelial growth factor production, which leads to reducing angiogenesis [47].

### 1.6. Phytochemicals

Diet is considered a well-established risk determinant in the development of GI cancer [48]. Following a rich phytochemical diet abundantly found in fruits, vegetables, nuts, and whole grains possesses several health protective benefits [49]. Phytochemicals in food science refer to a variety of plant ingredients which have different structures capable of promoting health [50]. Phytochemicals, known as secondary metabolites, are non-nutritive bioactive chemical compounds produced by plants [51]. They are called non-nutritive since they are synthesized by plants only in specific cells and not by the energy metabolism nor by the catabolic or anabolic metabolisms [52]. So far, 10,000 phytochemicals have been identified, including pre- and pro-biotics, polyphenols, carotenoids, steroids, and thiosulfate, while many remain unknown [53]. Phytochemicals are important for plant metabolism as they repel pests and sunlight and regulate plants growth [54]. Recently, phytochemicals have emerged as modulators of critical cellular signaling pathways and health improvement [55].

Phytochemicals have multiple health benefits for metabolic disorders such as cancer, cardiovascular disease, neurodegenerative diseases, and obesity [56]. Plants with a higher concentration of phytochemicals play a role in protection against free radical damage [57]. Research and clinical studies have postulated the anti-carcinogenic effects of phytochemicals, including their inhibiting of mitosis, inducing apoptosis, and enhancing the excretion of carcinogens [58]. In addition, phytochemicals possess both antioxidant and anti-inflammatory activities, where they interfere with several proinflammatory mediators [59].

### 1.7. Metabolism of Phytochemicals

Phytochemicals show variations in metabolism and deposition due to the variability in absorption, distribution, and excretion of phytochemical pharmacokinetics [60]. Examples of variational sources of phytochemicals include phase 1 metabolism in the liver where hydroxylation of phytochemicals can occur, resulting in novel secondary oxidation products [61]. Another example is metabolism by gut bacteria where phytochemicals undergo reduction, dehydroxylation, and demethylation, resulting in more biologically active metabolites [62]. In general, most phytochemicals present as glycosides or other conjugates in plant food, which means hydrolyzation is an essential process for absorption [63]. The hydrolysis could be achieved either by gut bacterial β-glucosidases in the colon or lower small intestine or by brush border membrane bound β-glucosidases [64]. After absorption, aglycones undergo extensive metabolism in the liver or gut epithelium with multiple compounds being conjugated by sulfotransferases (SULT) and UDP-glucuronosyltransferases (UGT) with glucuronic acid, glutathione, or sulfate, being potentially excreted in the bile or urine [65]. Compounds that are re-excreted through bile duct are deconjugated through bacterial β-glucuronidase, where they undergo enterohepatic recycling [64].

### 1.8. Search Strategy and Selection Criteria

Medline, Scopus, and PubMed were searched for papers published from 2000 using the search terms “phytochemical”, “phytochemicals AND cancer”, “phytochemicals AND colon cancer”, “phytochemicals AND GI cancer”, “phytochemicals subclasses AND cancer”, “carotenoid AND GI cancer”, “polyphenol and cancer”, ”prebiotics AND probiotics AND GI cancer”, and “phytochemical AND metabolism”. The search yielded approximately 6000 articles, and for this review, 237 articles were selected and analyzed.

## 2. Anti-Cancerous Effects of Selected Phytochemicals

### 2.1. Carotenoids

Carotenoids are pigments found in plants, bacteria, algae, and fungi [66]. The family of carotenoids (tetraterpenes) contains 500 compounds, 50 of which exhibit provitamin A activity [67]. While only 40 carotenoids have been identified in the human diet, human blood and tissue contain 20 carotenoids [68]. Carotenoids are well recognized for their antioxidant activities, regulation of cellular growth, immune response, and modulation of gene expression [69]. Pre-dominant carotenoids include lutein, lycopene, and β-carotene, which are abundantly found in egg yolk, tomato, and carrot [70].

#### 2.1.1. Lutein

**Figure 2:**
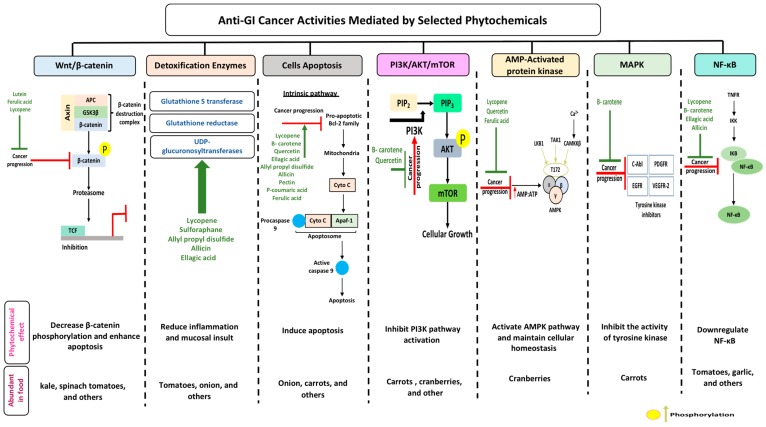
Phytochemicals as anti-GI cancer agents: mode(s) of action, aberrant signaling pathways (Wnt/β-catenin, detoxification enzymes, cellular apoptosis, PI3K/AKT/mTOR, AMPK, MAPK, and NF-κB), and pathway components targeted by phytochemicals (highlighted in green). Phytochemicals have a wide range of anti-cancerous actions through which one could target multiple mechanisms. These phytochemicals can enhance or suppress (green and red lines, respectively) the mechanisms through several activities. (see text for detailed mode(s) of action for phytochemicals mentioned).

Lutein in an abundant fat-soluble xanthophyll with a singular molecular formula (C_40_H_56_O_2_) [71]. It is found abundantly in egg yolk, oranges, yellow fruits, and green leafy vegetables [72]. Lutein is one of the two carotenoids that accumulates in fovea in the human retina [73]. It is a major constitute of macular pigment which is responsible for fine feature vision [74]. Recently, lutein has gained public health attention due to its putative role in protection against degenerative eye conditions and cancer [75]. A study performed on a Korean population showed an association between dietary lutein and the risk of colorectal cancer [76]. Lutein has considerable antioxidant function, which regulates apoptosis [77]. Administration of lutein in animal models has been observed to decrease the concentration of K-ras and AKT in tumors, resulting in cell cycle arrest [78]. Mice treated with lutein have been found to significantly inhibit aberrant crypt foci (ACF) development in the colon, reducing cellular proliferation [79]. Additionally, administration of lutein has been observed to reduce β-catenin concentration, hyperplasia, and adenocarcinoma in colonic samples [80]. It also acts as an effective blocking agent by reducing the concentration of specific protein-like β-catenin involved in cellular proliferation and apoptosis (Figure 2) [81]. Moreover, lutein plays a role in reducing reactive oxygen species and oxygen radicals while enhancing DNA damage repair (Table 1) [82].

#### 2.1.2. Lycopene

Lycopene is a lipophilic pigment and the main component of-red colored fruits and vegetables such as tomatoes [83]. Lycopene is structurally similar to β-carotene with the molecular formula C_50_H_56_, a hydrocarbon chain, and no functional groups [84]. The concentration of lycopene in tomatoes ranges 0.9 to 9.27 mg/g [85]. Lycopene is a potent antioxidant which can counteract reactive oxygen species like peroxyl radicals [86]. The expression of lycopene’s antioxidant activity is due to (i) the detoxification process through the production of enzymes like glutathione peroxidase (GPx), glutathione-S-transferase (GST), and glutathione reductase (GR); (ii) the inhibition of cytochrome P450 2E1, which is critical for the conversion of xenobiotics in cancer; and (iii) the suppression of carcinogen progression (Figure 2) [87]. In addition, lycopene exerts both anti-inflammatory and anti-cancer activity specifically against colorectal cancer [88]. Administration of lycopene using gold nanoparticles as a vehicle has been found to reduce the expression of pro-caspase 3, 8, and 9 and enhance Bcl-2-associated X protein (BAX) expression, thus enhancing the apoptotic pathway [89]. A one-day cultured colon cancer cell with 10 μm of lycopene showed a reduction in cellular growth by reducing the expression of Hmg Co-A reductase and enhancement in Ras translocation from the plasma membrane to cytosol [90]. Lycopene is reported to inhibit the expression of NF-κB and c-Jun N-terminal kinases (JNK), which (i) leads to a decreased tumor necrosis factor α (TNF-α), interleukin-1 (IL-1), and IL-6 and (ii) inhibits the expression of cyclooxygenase 2 (COX-2) and NO production (Table 1) [91]. In a gastric-induced carcinogens model, lycopene has been found to block the activity of carcinogenic cells through the upregulation of a reduced glutathione (GSH) dependent hepatic detoxification system, thus protecting cells from oxidative damage [92].

#### 2.1.3. β-Carotene

β-carotene, a core member of the carotenoid family, has been well documented for its natural antioxidant, anti-inflammatory, and anti-cancerous activity in recent years [93]. Carrot (*Daucus carota* L) is a popular root vegetable and an important dietary source of carotenoids [94]. The chemical composition of carrots includes moisture (86%), protein (0.9%), fat (0.2%), carbohydrate (10.6%), fiber (1.2%), total ash (1.1%), Ca (80 mg/100 g), p (53 mg/100 g), and Fe (2.2 mg/100 g) [95]. Carrots have been reported to inhibit the formation of neoplastic tumors in colonic cancer rat models, affecting the composition of low abundant gut microbiota like *Lactobacillus reuteri* [96]. Human cancer cells treated with 50 and 100 μg/mL or pentane fraction and 1:1 pentane: diethyl ether fraction have shown an inhibition in cellular proliferation due to induced sub G1 phase accumulation and enhanced apoptotic cell death [97]. Additionally, cancerous cells treated with carrot oil extract have reported an increase in BAX and P53 levels and a decrease in Bcl-2 levels (Table 1) [98]. Studies have shown that carrots inhibit cellular proliferation and induce apoptosis and cellular arrest through the suppression of the MAPK/ERK and PI3L/AKT pathways (Figure 2) [99]. A study has reported that consumption of carrots is more effective in the prevention of gastric cancer in people at risk of the disease (those with a family history of gastric cancer) compared to other people. This suggests that following a healthy lifestyle could prevent the development of gastric cancer in people with higher risk [100].

### 2.2. Proanthocyanidins

Proanthocyanidins, also known as condensed tannins, result from flavanol condensation [101]. They are abundantly found as polymers and oligomers in fruits, barriers, seeds, leaves, and flowers [102]. Recent interest in proanthocyanidins has been stimulated due to their potential health benefits which arise mainly from their antioxidant activity [103]. The effectiveness of proanthocyanidins are determined by gut microbiome composition [104]. Additionally, they have anticancer properties via the reduction of tumor development by inducing apoptosis or inhibiting cellular proliferation [105].

#### 2.2.1. Quercetin

The cranberry (*Vaccinium macrocarpon*) is a fruit which has been used as a functional food due to its health benefits [106]. It is a rich source of polyphenols, which exerts anti-inflammatory, antiviral, antibacterial, antioxidant, anticarcinogenic, and antimutagenic activities [107]. It has a complex and rich phytochemical composition, consisting predominantly of A-type procyanidins (PACs), flavan-3-ols, anthocyanins, ursolic acid, quercetin, and benzoic acid [108]. Recently, the cranberry has received attention as a result of its effects related to lowering the risk of cancer [109]. Animal studies have reported the chemoprotective effect of cranberry to suppress the growth of several types of cancer cells, including colon, lung, prostate, oral, and ovarian [110]. Administration of 20% cranberry juice in water to rat models demonstrated a reduction in the total number of ACF [111]. Cranberry extracts have been reported to reduce proinflammatory interleukins and C-reactive protein [112]. APC^min/+^ mice fed with 20% (*w/w*) freeze dried whole cranberry powder for 12 weeks showed a significant prevention of intestinal tumor formation (33.1%) due to induced cellular apoptosis and reduced cellular proliferation [113]. Also, it is reported that cranberry consumption inhibits the activation of the PI3K, AKT, and COX-2 signaling pathway (Table 1) [114]. Administration of cranberries has shown an activation in the AMPK pathway which helped maintain cellular homeostasis [115].

#### 2.2.2. Ellagic Acid

The bilberry (*Vaccinium myrtillus* L) is a rich natural source of anthocyanins [116]. Total anthocyanin content in the bilberry ranges from 300–700 mg/100 g [117]. It is classified by the American Herbal Products Association as a class 1 herb, which means it can be safely consumed when used appropriately [118]. Ellagic acid is a phenolic compound found in bilberry extracts which has potent antioxidant properties and can chelate metal ions and scavenge free radicals [119]. Treatment of rats’ hepatocyte primary culture with bilberries has shown a protective effect against oxidative damage [120]. Bilberries have been reported to induce phase II xenobiotic detoxification enzymes, which are critical for cancer prevention [121]. Additionally, bilberry-rich extracts have been observed to inhibit the growth of colon cancer cells but to not affect normal colon cells, thus suggesting a possible protective effect against cancer [122]. Rats with genetic colon adenoma fed concentrated bilberry extract (10% *w*/*w*) have shown a significant reduction in intestinal adenoma by 15–30% [123]. In a pilot study on 25 patients with colorectal cancer who were given bilberry extract for 7 days, the results showed a significant reduction in tumor cellular proliferation by 7% compared to the results before bilberry administration [124]. Treatment of human monocytic THP-1 cells with bilberry extract showed reduction in pro-inflammatory gene expression, interferon γ (IFN-γ), and cytokine secretion [125]. Moreover, bilberry extract exerts the ability to induce apoptosis and arrest growth in GI cancer (Figure 2) [126]. Bilberry extract has been reported to diminish topoisomerase catalytic activity in colon carcinoma cells, showing a protective DNA effect [127].

### 2.3. Organosulfur Compounds

Organosulfur compounds are sulfur-containing organic compounds with beneficial anti-inflammatory, antioxidant, and anti-cancerous effects [128]. Animal and epidemiological studies have shown that administration of organosulfur compounds reduces the risk of colorectal cancer through the induction of mitotic arrest and apoptosis [129,130]. Garlic, onion, asparagus, and cruciferous vegetables are abundant in organosulfur compounds.

#### 2.3.1. Allicin

Attention has been given recently to garlic due to its high content of flavonoids and organosulfur compounds like allicin [131]. Worldwide garlic (*Allium sativum*) has been frequently used as a dietary botanical supplement [132]. Ally sulfur compounds like allicin found in garlic (1% of garlic’s dye weight) seems to be responsible for the beneficial effects of garlic [133]. Animal studies have shown that administration of garlic reduces the formation of ACF [134]. The mechanisms by which garlic inhibits the growth of carcinogen cells include reduction of DNA adducts, regulation of cellular arrest, activation of metabolizing detoxification enzymes, and induction of differentiation and apoptosis [135,136]. Organosulfur compounds present in garlic have shown potential for an anti-cancer drug by the modulation of epithelial growth factor receptor (EGFR), which plays a role in cell division [137]. Results obtained from an induced colitis mouse model have shown that administration of diallyl disulfide extracted from garlic is able to prevent the development of colitis-induced colorectal cancer [138]. In addition, garlic has been observed to prevent prolonged inflammation in mice, which supports the chemoprotective effect of garlic in CRC [139]. Moreover, consumption of garlic suppresses the activity of NF-κB by inhibiting phosphorylated P65 translocation (Figure 2) [140]. In xenograft nude mice, administration of S-allylmercaptocysteine (SAMC) in combination with rapamycin (a macrolide compound) was found to enhance anticancer ability by suppressing tumor growth and inducing apoptosis (Table 1) [141]. Administration of aged garlic extract in rat tumor models has been shown to attenuate colon tumor progression effectively by reducing cellular proliferation through the attenuation of NF-κB activity [142]. A meta-analysis study has indicated that the consumption of garlic is associated with reduced gastric cancer with a 95% confidence interval and a 0.53 odd ratio [143].

#### 2.3.2. Allyl Propyl Disulfide

Chemical groups found in onions such as flavonoids, alk(en)yl cysteine sulfoxides (ACSOs), and allyl propyl disulfide are associated with the health benefits of onions [144]. The consumption rate of onion (*Allium cepa* L.) has increased worldwide, leading to an increase in the national production of onion by 25% over the last decade [145]. Compounds from onions have been reported to have multiple health benefits, including having antiplatelet, anticarcinogenic, and antithrombogenic activities [146]. Onion extracts have been reported to significantly induce apoptosis and reduce cellular proliferation in colorectal cancer [147]. An in vivo study has indicated that administration of onion in a hyperlipidemic colorectal cancer model plays a similar role to capecitabine in a colorectal cancer model without hyperlipidemia by inhibiting CRC and reducing hyperlipidemia [148]. Human cancer adenocarcinoma cells treated with 200 μm Se-methyl-L-selenocysteine (MSeC) for 24 h have been found to trigger 80% apoptosis in cells through endoplasmic reticulum stress rather than reactive oxygen species stress (Table 1) [149]. The benefit of onions is not limited to reducing or treating GI cancer but also to detecting cancer. One study used carbon nano onion films to develop a capacitive immunosensor for a CA19-9 cancer biomarker detector which succeeded in detecting CA19-9 in whole lysate colorectal adenocarcinoma using the sensor combined with information visualization methods [150].

#### 2.3.3. Asparagusic Acid

Asparagus species are native medical shrubs which have beneficial medical properties and which belong to the Liliaceae family [151]. Major bioactive compounds found in asparagus include steroidal saponins, asparagusic acid, vitamins (A, B_1_, B_2_, C, E, Mg, P, Ca, and Fe), folic acid, asparagine, tyrosine, arginine, essential oils, tannin, resin, and flavonoids. The health properties of asparagus include anti-microbial, antioxidant, and cytotoxic activities [152]. Asparagus extracts have illustrated a potent cytotoxic effect against colorectal cancer [153]. Treatment of Myeloid-derived suppressor cells (MDSCs) with asparagus polysaccharide have shown a significant increase in apoptosis through intrinsic pathways and a significant decrease in cellular proliferation [154]. Old stems of asparagus (SSA) tested on colon cancer cells have been found to suppress cellular viability and block cellular migration and invasion through Rho GTPase signaling pathway modulation [155]. In human colon adenocarcinoma, methanolic extracts from white asparagus have demonstrated TRAIL death receptor pathway activation leading to the activation of caspase-8 and caspase-3, and, finally, to cell death. In addition, asparagus extracts have been seen to inhibit cellular pro-inflammatory mediators like MMP7, MMP9, and TNF-α [156].

#### 2.3.4. Sulforaphane

Cruciferous vegetables refer to those which belong to the Brassicaceae family and include cabbage, broccoli, and Brussel sprouts [157]. This family is known for the glucosinolate, a sulfur-containing compound synthesized endogenously in plants derived from amino acid and glucose residues [158]. Upon cellular rupture through vegetable consumption, glucosinolates are hydrolyzed by endogenous enzymes and produce potential compounds such as thiocyanates and nitriles [159]. Cruciferous vegetables contain several phytochemical compounds such as sulforaphane. Studies have shown the beneficial effects of cruciferous vegetables which have helped inhibit the development of GI cancer [160]. In vivo and in vitro studies have demonstrated the ability of cruciferous vegetables to defend healthy cells against radiation and chemically-induced carcinogenesis [161]. Additionally, these vegetables have been shown to inhibit cellular proliferation, migration, and survival of tumor cells [162]. Cruciferous vegetables demonstrate antioxidant activity as they widely show a protective effect against oxidative stress through the depletion of glutathione [163]. Additionally, these vegetables induce acute oxidative stress through the inhibition of P38 MAPK, which inhibits Nrf2-Keap 1 dissociation (Table 1) [164]. Cruciferous vegetables guard against colorectal cancer through several mechanisms: (i) the modulation of detoxification enzymes (Figure 2), (ii) the induction of cellular apoptosis, and (iii) the controlling of cancer cellular growth through cell cycle arrest [165,166,167]. A meta-analysis study has shown that cruciferous vegetables significantly reduce the risk of gastric and colorectal cancer by 19% and 8%, respectively [168].

### 2.4. Other Phytochemicals

The following four phytochemicals did not fit under any of the above categories, and, therefore, due to their beneficial anticancer effects, we decided to give them a section of their own. 

#### 2.4.1. Pectin

Pectin is a natural polysaccharide derived from the cell walls of plants like citrus fruits and apples consisting of a linear chain of and α-(1–4) linked D-galacturonic acid residues, with some of the galacturonic acid carboxyl group being methyl esterified [169]. Depending on the percentage of esterification, high methoxy pectin (more than 50%) or low methoxy pectin (less than 50%) can be formed [170]. Pectin has been proven to have multiple biological effects like the reduction of cholesterol, lipid, insulin, and sugar level in the body. In addition, pectin exerts anticancer activities [171]. Pectin has been used in the colon drug delivery carrier system as well as being conjugated with other drugs like cisplatin, thus enhancing the blood circulation of the drug in mice models [172,173]. Using pectin as a new anticancer oral drug delivery system will enhance antitumor efficacy and reduce the risk of systemic toxicity in colon cancer [174].

Gastric cancer cells treated with low molecular weight citrus pectin (LCP) have been found to suppress adhesion, aggregation, and metastasis of cancer cells [175]. Moreover, LCP decreases the activity of premetastatic protein GAL-3 resulting in the promotion of caspase-mediated apoptosis and inhibition of tumor cell growth [176]. Plant-based non-digestible carbohydrates like pectin show potential in reducing the risk of colorectal cancer through the inhibition of GAL-3 protein expression, thus enhancing apoptosis and inhibiting cancer cells migration [177]. Pectin extracted from potatoes has demonstrated a significant reduction in cellular proliferation of colon cancer cells through the suppression of intercellular adhesion molecule 1 (ICAM 1) expression [178,179].

#### 2.4.2. Curcumin

Curcumin is an active phytochemical compound originating from the rhizomatous herbaceous perennial plant of the ginger family (Curcuma longa) [180,181]. Recently, curcumin has given more attention due to its multiple health benefits, especially with regard to the management of degenerative and inflammatory eye conditions [182]. Greater attention has been given to cancer and curcumin in recent years (21,098 articles have been published in PubMed in the last 10 years). Due to this huge number of articles, we decided to add the most important points in Table 1. In addition to this, we cite four articles we evaluated which discuss the correlation between GI cancer and curcumin and which have been published in the last two years [183,184,185,186].

#### 2.4.3. p-Coumaric Acid

p-Coumaric acid is an aromatic phenolic compound found in navy beans (*Phaseolus vulgaris*), which are an economically and nutritionally important food crop worldwide [187]. Navy beans are rich in protein, carbohydrates, minerals, dietary fibers, vitamins, and many other polyphenolics structurally similar to p-coumaric acid [188]. They exhibit several beneficial health effects like anticancer and anti-microbial infection properties and anti-human immunodeficiency virus effects [189]. Rats fed with navy beans have shown a significant reduction in colon adenocarcinoma compared to controls. This significant reduction can be attributed to a (i) high level of butyrate production in the distal colon and (ii) a more controlled appetite, which reduces body fat significantly [190]. Dietary intervention in CRC survivors for four weeks has shown that the consumption of navy bean reduces the risk of CRC and other chronic conditions. In addition to this, results have measured how serum inflammatory markers can provide the chemoprotective effects of navy beans against CRC [191]. Navy beans and their metabolites (Table 1) have been implicated in the protection of CRC through the modulation of major metabolic pathways like lysine, sterol, amino and inositol, and fatty acid metabolism. Dietary intake of navy beans (35 g/day) has been found to increase the abundance of several amino acids in stools, showing a protective effect against CRC through the inhibition of cellular progression, reduction of oxidative stress, and induction of cellular apoptosis [192,193]. A study performed on 16 people (seven non-cancer subjects and nine CRC survivors) has shown that the addition of 35 g cooked navy bean powder in meals for 28 days is able to provide chemoprevention effects against CRC [194].

#### 2.4.4. Ferulic Acid

Ferulic acid is a bioactive component found in rice bran [195]. Rice bran is one of the byproducts produced by the milling process of rice [196]. Great attention has been paid to rice bran recently due to its high nutritional value, potential to improve heath, low cost, and availability [197]. Bioactive and phytochemical compounds of rice bran, such as ferulic acid, have been reported to possess anti-inflammatory, anti-diabetic, anticancer, and antioxidant activities [198]. The oil present in rice bran contains specific bioactive compounds that are considered to have a cardiac friendly chemical profile [199]. Studies have shown that rice bran intake modifies intestinal microbiota, which helps to prevent CRC [200]. Additionally, rice bran exhibits antitumor activities through the modulation of multiple pathways like inhibition of oxidative stress and induction of apoptosis [201]. In vitro cancer studies have shown that rice bran and its bioactive compounds (Table 1) have been shown to inhibit carcinogenesis by suppressing chronic inflammation, blocking cell signaling, inhibiting proliferation, scavenging free radicals, and activating detoxification enzymes (Figure 2) [202,203,204]. The consumption of rice bran has been found to reduced the adenoma burden in APC^min^ mice through the modulation of adiponectin which was observed to be significantly altered, thus playing a role in tumor suppression [205]. Administration of inositol hexaphosphate extracted from rice bran has been reported to significantly suppress β-catenin and COX-2 expression, which could inhibit the development of CRC [206,207]. In addition, human colorectal adenocarcinoma HT-29 cells treated with inositol hexaphosphate have been shown to induce apoptosis through the upregulation of BAX, caspase-3, and caspase-8 expression, and through the downregulation of Bcl-xl [208].

**Table 1:** Representive Phytochemicals and Their Underlying Anti-Cancerous Effects.

Phytochemical Subclass	Phytochemical and Structure	Dietary Source	Conversion Reaction	Metabolites Produced	Mechanism of Action	Model Used		References
						In Vivo	In Vitro	
**Carotenoids**	Lutein 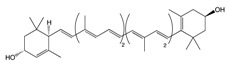	Egg yolk, kale, spinach, parsley, and peas	Oxidation	3′-dehydro-lutein	* Reduces slightly the risk of colorectal cancer * Reduces the risk of colorectal neoplasms in women * Inhibits the growth of carcinoma cells * Decreases the concentration of AKT expression which reduces cellular proliferation * Decreases β-catenin concentration thus enhancing the apoptotic pathway * Regulates miRNA expression through DICER 1 activity * Enhances DNA damage repair * Induces humoral and cell mediated-immune response * Scavenges against oxygen radicals * Quenches reactive oxygen species * Activates MAPK pathway through MAP3K9 interaction * Protects against the formation of colonic aberrant crypt foci	* Sprague-Dawley rats.	* Human normal colon epithelial cells * Human colon adenocarcinoma cells	[74,78]
	Lycopene 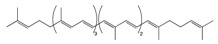	Tomato, guava, papaya, grapefruit, and watermelon	Auto-oxidation Radical-mediated oxidation	Apo-10′-lycopenoids	* Suppresses the progression of carcinogenesis through the inhibition of DNA synthesis * Inhibits cell invasion, metastasis, and angiogenesis * Reduces cell migration capacity * Downregulates AKT, NF-κB, MMP-2, MMP-7, and MMP-9 * Decreases β-catenin concentration * Reduces pro-inflammatory mediators and enzymes such as TNF-a and COX-2, respectively * Prevents oxidative damage through scavenging oxygen free radicals * Suppresses the expression of cyclin D1 and PCNA proteins * Inhibits the formation of colonic ACFs * Stimulates the activity of enzymes such as glutathione reductase, glutathione peroxidase, and glutathione S-transferase * Enhances apoptotic pathway * Activates MAPK signaling gene * Upregulates p21 cell cycle inhibitor protein	* Induced-colitis rat models * Sprague-Dawely rats * Fischer 344/NSIc rats	* HT-29 cell lines	[86,89]
	β-Carotene 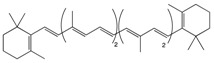	Carrot	Oxidation	Falcarindiol 6-methoxymellein	* Inhibits the formation of neoplastic tumors * Reduces the number of polyps in the colon * Inhibits pleiotropic cytokines and the NF-κB pathway * Reduces the formation of macroscopic neoplasms by targeting low abundant gut microbiome * Inhibits cellular proliferation through MAPK/ERK and PI3K/AKT pathway inhibition * Enhances p53-dependent apoptosis pathway	* Azoxymethane (AOM) treated rats	* HT-29 cells * HCT 116 cells * CCD-33Co cells	[97,98,99]
**Pro-anthocy-anidins**	Quercetin 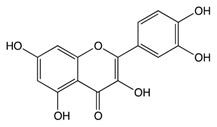	Cranberry	Sulfation Conjugation	3-(4hydroxyphenyl) -propionic acid hippuric acid catechol-O-sulfate	* Reduces small intestine tumor formation * Reduces inflammatory responses when consumed with fiber * Reduces tumor incidence, multiplicity, burden, and average tumor volume * Reduces colonic inflammatory cytokine expression such as IFN-γ and TNF-α * Inhibits the activation of the PI3K, AKT, and COX-2 signaling pathway * Inhibits cancer cell proliferation and tumor growth * Inhibits VEGF, MMP-2, and MMP-9 expression * Inhibits the incidence of AOM-induced ACF * Induces cellular apoptosis * Increases the number of colonic goblet cells and MUC 2 production * Increases caecal short fatty acids concentration	* Apc(min/+) mice * Male weanling rats	* HCT116 cell lines * HT-29 cell lines * Cancer cell line encyclopedia (CCLE)	[108,109]
	Ellagic Acid 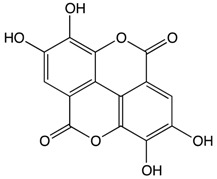	Bilberry	Glucuronidation O-methylation	Peonidin-3-galactoside	* Reduces the expression of proinflammatory cytokines * Reduces inflammation and tumor development * Inhibits cellular proliferation * Inhibits the formation of colonic ACFs * Suppresses the activity of topoisomerase I and II which reduces DNA damage * Induces cellular apoptosis through NF-κB inhibition * Protective activities against colorectal cancer	* Female Balb/c mice	* Intraepithelial neoplasia * HCT-116 cell line	[120,121]
**Organosulfur Compounds**	Allicin 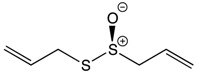	Garlic	Oxidation Hydrolysis	Allyl methyl sulfide (AMS) Allyl methyl sulfoxide (AMSO) Allyl methyl sulfone (AMSO_2_)	* Inactivates NF-κB localization by inhibiting glycogen synthase kinase 3 (GSK-3) which prevent colitis-induced colorectal cancer * Suppresses cellular proliferation and tumor growth * Induces colon cancer cell apoptosis * Anticancer activity against colorectal cancer through the modulation of epithelial growth factor receptor (EGFR) * Activates antioxidative transcriptor Nrf2	* Xenograft nude mice	* HCT-116 cell line	[135,136]
	Allyl propyl disulfide 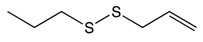	Onion	Reduction	Quercetin 3,4‘-diglucoside Quercetin 4‘-glucoside	* Reduces cellular proliferation * Reduces migration rate of cancer cells * Reduces tumor growth rate in colorectal cancer * Induces cellular apoptosis * Induces cell cycle arrest at G2/M phase		* Caco-2 cell line * SW620 cell line	[146,147]
	Asparagusic acid 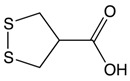	Asparagus	Sulfation	Asparagus polysaccharide dimethyl sulfide	* Cytotoxic effect against human colon cancer cell greater than 5-FU * Reduces cellular proliferation * Inhibits cell motility and cellular growth by targeting Rho GTPase signaling pathway * Induces intrinsic apoptosis through toll-like receptor 4 * Enhances the expression of BAX and Caspase 9		* HCT-116 cell line * Caco-2 cell line	[152,153]
	Sulforaphane 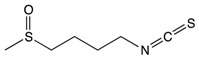	Broccoli, cabbage, Brussels sprout, and cauliflower	Hydrolysis	Thiocyanates Isothiocyanates Epithionitrile nitrile	* Reduces the risk of adenomatous polyps * Prevents colorectal cancer through miRNA modulation * Protects against Barrett’s esophagus * Induces apoptosis and cellular arrest * Induces detoxification enzymes * Cytoprotective effect through the induction of Nrf2 * Scavenges against free radicals		* Squamous cell carcinoma	[164,165]
**Other Phytochemicals**	Pectin 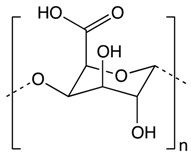	Apples, plums, oranges, and gooseberries	Colonic fermentation	Butyrate	* Inhibits cancer cell metastasis of gastrointestinal cancer * Inhibits colon cancer cell proliferation by downregulating ICAM1 expression * Induces apoptosis by downregulating Bcl-xL and Cyclin B * Modulates the expression of signature miRNA * Delivers oral drugs for colon cancer treatment	* BALB/c mice	* HCT116 cells * Caco-2 cell line	[169,170]
	Curcumin 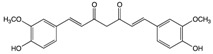	Ginger	Hydrolysis	Curcumin glucuronide Curcumin sulfate	* Suppresses tumor growth by suppressing PPARγ pathway * Prevents cellular proliferation * Induces cellular apoptosis * Upregulates the expression of Caspase-3, cytochrome C, and BAX		* Cancer stem-like cells (CSC)	[183,184]
	p-Couramic acid 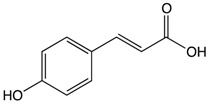	Navy beans	Hydrolysis	N-methylpipecolate 2-aminoadipate Piperidine Vanillate	* Reduces oxidative stress * Reduces the number of colonic aberrant cypt foci * Anti-tumor activity against colorectal cancer * Increases the abundance of amino acids, phytochemicals, and lipids in stool * Induces cellular apoptosis	* FVB/N mice		[189,190]
	Ferulic acid 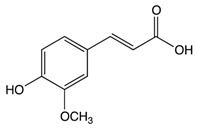	Rice bran	Colonic fermentation	Tryptophan α-ketoglutarate γ-tocopherol/β-tocopherol γ-tocotrienol	* Inhibits cellular proliferation, cell cycle progression, and tumor growth * Decreases β-catenin and COX-2 in colon tumors * Increases the production of SCFAs * Induces nitric oxide synthase expression, Caspase-3 activation, and NF-κB pathway * Induces cellular apoptosis and lipid peroxidation * Scavenges free radicals * Modifies the composition of intestinal microbiota	* APC (min) mice	* Caco-2 cells * HAT-29 cells	[201,202]

## 3. Conclusions

Phytochemicals are biologically active compounds found in plants. They have illustrated anticancer activity against gastrointestinal cancer through the modulation of several mechanisms like inducing apoptosis, inhibition of oxidative stress and cellular progression, and blocking cellular signaling.

### 3.1. Challenges with Studying Phytochemicals

Although a lot of efforts have been spent to study the activities of phytochemicals as an anticancer agent, lots of limitations are linked to these studies. Firstly, the assessment of most of the studies performed has depended on an in vitro evaluation [209]. Most of the in vitro studies have been based on ethnopharmacological information where the selection of plants or phytochemicals occurs first, and then molecular-based approaches are used to emphasize the similarity between phytochemicals and approved drugs by comparing molecular structure and protein target cites and linking them to the potential health benefits [210,211]. These approaches are designed to study the anticancer effects of phytochemicals on specific phenotypes, which makes it difficult to analyze and generalize the health effects on the human body [212]. Secondly, lots of attention has been given to the positive effects of selective phytochemicals without any mention of the negative effects of other phytochemicals which could be potential carcinogens or tumor promoters [213].

#### 3.1.1. Estimated Consumption Level of Phytochemicals

Phytochemicals are non-established nutrients with significant health promotion and protective effects [214]. When it comes to specifying the recommended phytochemical intake, several points need to be recognized: (i) the health benefits associated with phytochemicals cannot be attributed to a specific phytochemical compound, (ii) different biological activities make it hard to select a specific key biological role for phytochemicals and generalize this to the whole population, and (iii) there is no assigned disease or impaired function linked to one or any of the members of the phytochemical group [215]. Worldwide, efforts have been made to create an optimal consumption level for selected phytochemicals. A US study on twenty-six healthy participants showed that consumption of 13–22 g dietary fiber per day for 3 weeks reduces cholesterol levels by 7% [216]. The consumption level of phytochemicals varies between populations. For example, Italians consume 14.3 mg/day of total carotenoids while Americans consume 6.6–10.5 mg/day for men and 5.7–10.4 mg/day for women [217,218]. Studies have reported that consumption of lutein of up to 20 mg/day is safe and does not contribute to diverse side effects [219]. Limited information is available regarding the recommended dosage of phytochemicals due to many challenges. Firstly, there is limited information related to their bioavailability and heterogenicity. Secondly, there is limited information regarding the effects of food processing and dietary consumption on the effectivity of these flavonoids. Thirdly, differences in the absorbability of phytochemicals have been observed depending on the source of food [220].

#### 3.1.2. Could Phytochemicals be Carcinogenic

Recently, more attention has been paid to secondary metabolites (phytochemicals) found in fruits and vegetables [221]. This has led to an increase in the consumption rate of these natural substances either in their natural form or as a supplement for medical purposes [222]. Many cancer patients use phytochemical supplements in combination with prescribed cancer treatments [223]. Despite the popular usage of phytochemicals, limited information is available regarding their toxicity and safety levels [224]. A number of phytochemicals found in people’s food and seasonings have shown potential carcinogenic effects [225]. One example is capsaicin, a principle pungent component belonging to the genus *Capsicum* which is responsible for the hotness or intensity of chili peppers [226]. Multiple epidemiological studies suggest the protective role of capsaicin in cancer treatment [227]. Results from animal studies have reported the opposite, stating the carcinogenic effect of capsaicin [228]. These controversial results which have been obtained suggest caution when consuming these phytochemicals and that normalize and control studies are needed to prevent result discrepancies. Other examples of phytochemicals as potential carcinogens include cycasin, phytoestrogens, ptaquiloside, and safrole [229,230,231,232]. So far, no specific data are available to address the short- and long-term effects of phytochemical consumption and their potential as tumor promoters or carcinogens.

#### 3.1.3. Could Phytochemical Combinations Have Synergistic Effects

The consumption of phytochemicals triggers multiple selected mechanisms, as highlighted in Table 1. Studies have shown that administration of cancer treatments combined with phytochemicals may improve the prognosis of the disease, as it affects multiple pathways [233]. If we could trigger multiple mechanisms by combining the consumption of multiple phytochemicals, will this improve cancer manifestations? For example, administration of lycopene triggers five mechanisms, namely, it downregulates the NF-κB pathway and Wnt/β-catenin, upregulates MAP-mediated protein kinase, detoxifies enzymes, and induces apoptosis. Will the administration of carrots which activate the two other mechanisms not activated by lycopene (PI3K/AKT/mTOR and MAPK) improve the anti-cancerous activities mediated by these secondary metabolites (Figure 2)? Would this lead to a better outcome in which the action needed may be approached while the over-activation of mechanisms may be prevented?

Further research is needed to confirm the applicability of this suggestion, as well as the side effects of this combination, as there is little known about phytochemical/phytochemical interactions.

#### 3.1.4. Phytochemicals in Cancer Drug Delivery

Cancer treatment with conventionally formulated anti-cancer drugs possesses multiple limitations like low solubility in water, short half-life in the body, poor specificity, and poor oral administration suitability [234]. Studies have shown enhancement effects when using phyto-nanotechnology on cancer cell lines [235]. This technology offers promising solutions for drug delivery systems by combining a phytochemical-based drug with a synthetic drug and then introducing the combination into the body [236]. Using nanotechnology in drug delivery increases bioavailability, decreases toxicity, prolongs circulation time, and improve efficacy [237]. More research is needed to confirm the positive effects of using this technology.

### 3.2. Final Thoughts

Phytochemicals found in fruits and vegetables have multiple beneficial effects on GI cancer. A diet rich in phytochemicals could improve the prognosis of GI cancer. Generally, the combination of phytochemicals could enhance anticancer effects by triggering multiple mechanisms, but more research is needed to support this promising means of enhancing cancer prognosis and possibly prevention. More attention needs to be paid to studying the bacteria involved in the biotransformation of phytochemicals to their metabolites, which could potentially help predict the health status of humans. Overall, a diet rich in fruits and vegetables is recommended.

## Abbreviations

GI	gastrointestinal
CRC	colorectal cancer
APC	adenomatous polyposis coli
SMAD	small mothers against decapentaplegic
IL	interleukins
SCFA	short chain fatty acid
Wnt	wingless-related integration site
TNF	tumor necrosis factor
AMPK	adenosine monophosphate activated protein kinase
PI3K	Phosphoinositide 3-kinases
STZ	streptozotocin
NF-κB	nuclear Factor kappa-light-chain-enhancer of activated B cells
Bcl-2	B-cell lymphoma 2
NO	nitric oxide
SULT	sulfotransferases
ACF	aberrant crypt foci
UGT	UDP-glucuronosyltransferase
GPx	glutathione peroxidase
GST	glutathione S-transferase
GR	glutathione reductase
BAX	Bcl-2-associated X protein
JNK	c-Jun N-terminal kinases
GSH	reduced glutathione
COX-2	cyclooxygenase 2
Nrf2	nuclear factor erythroid 2-related factor 2
PACs	predominant A- type procyanidins
EGFR	epithelial growth factor receptor
TBARS	thiobarbituric acid reactive substances
ROS	reactive oxygen species
SAMC	S-allylmercaptocysteine
ACSOs	alk(en)yl cysteine sulphoxides
MseC	Se-methyl-L-selenocysteine
MDSCs	myeloid-derived suppressor cells
MMP	matrix metallopeptidase
ICAM	intercellular adhesion molecules
